# Beyond viral suppression: Quality of life among stable ART clients in a differentiated service delivery intervention in Tanzania

**DOI:** 10.1007/s11136-021-02889-z

**Published:** 2021-05-30

**Authors:** Nwanneka Ebelechukwu Okere, Veronica Censi, Clementina Machibya, Kathleen Costigan, P. Katambi, Giulia Martelli, Josien de Klerk, Sabine Hermans, Gabriela B. Gomez, Anton Pozniak, Tobias Rinke de Wit, Denise Naniche

**Affiliations:** 1 Department of Global Health, Amsterdam Institute for Global Health and Development, Amsterdam UMC, University of Amsterdam, Amsterdam, Netherlands; 2 Doctors with Africa (CUAMM), Test & Treat Project Shinyanga, Shinyanga, Tanzania; 3 Ngokolo Health Centre, Catholic Diocese of Shinyanga, Shinyanga, Tanzania; 4 Bugisi Health Centre, Catholic Diocese of Shinyanga, Shinyanga, Tanzania; 5 Department of Global Health and Development London School of Health and Tropical Medicine United Kingdom, London, UK; 6 Chelsea and Westminster Hospital NHS Foundation Trust, London, UK; 7 Barcelona Institute for Global Health, University of Barcelona, Barcelona, Spain

**Keywords:** Quality of life, FAHI, Wellbeing, Emotional, Social, Differentiated service delivery

## Abstract

**Background:**

With antiretroviral therapy, more people living with HIV (PLHIV) in resource-limited settings are virally suppressed and living longer. WHO recommends differentiated service delivery (DSD) as an alternative, less resource-demanding way of expanding HIV services access. Monitoring client’s health-related quality of life (HRQoL) is necessary to understand patients’ perceptions of treatment and services but is understudied in sub-Saharan Africa. We assessed HRQoL among ART clients in Tanzania accessing two service models.

**Methods:**

Cross-sectional survey from May–August 2019 among stable ART clients randomly sampled from clinics and clubs in the Shinyanga region providing DSD and clinic-based care. HRQoL data were collected using a validated HIV-specific instrument—Functional Assessment of HIV infection (FAHI), in addition to socio-demographic, HIV care, and service accessibility data. Descriptive analysis of HRQoL, logistic regression and a stepwise multiple linear regression were performed to examine HRQoL determinants.

**Results:**

629 participants were enrolled, of which 40% accessed DSD. Similar HRQoL scores *[mean (SD), p-value]*; FAHI total [152.2 (22.2) vs 153.8 (20.6), *p* 0.687] were observed among DSD and clinic-based care participants. Accessibility factors contributed more to emotional wellbeing among DSD participants compared to the clinic-based care participants (53.4% vs 18.5%, *p* =  < 0.001). Satisfactory (> 80% of maximum score) HRQoL scoring was associated with (OR [95% CI], *p*-value) being male (2.59 [1.36–4.92], *p* 0.004) among clinic participants and with urban residence (4.72 [1.70–13.1], *p* 0.001) among DSD participants.

**Conclusions:**

Similar HRQoL was observed in DSD and clinic-based care. Our research highlights focus areas to identify supporting interventions, ultimately optimizing HRQoL among PLHIV.

**Supplementary Information:**

The online version contains supplementary material available at 10.1007/s11136-021-02889-z.

## Background

Access to effective antiretroviral therapy (ART) has contributed to an increased number of people living with HIV (PLHIV) being virally suppressed and living longer [1–4]. However, the physical consequences of long-term exposure to ARVs have yet to be fully elucidated and evidence associates PLHIV on ART with an increased risk of cardiovascular disease, liver disease, and various malignancies [5–8]. While PLHIV report significantly lower health-related quality of life (HRQoL) when compared to the general population in high-income countries (HIC) [9], HRQoL among PLHIV is understudied in low and middle-income countries (LMIC), especially in sub-Saharan Africa (SSA). With an increasing number of PLHIV on ART who are aging in SSA, monitoring of HRQoL becomes a priority in this setting [10, 11].

HRQoL is a multidimensional concept depicting an individual’s subjective perception of current health status and outlook of the future [12, 13]. HRQoL studies assess individuals’ perception of their health and how it affects or is affected by other aspects of life [13]. Among PLHIV, ART impacted HRQoL positively, especially in LMIC when ART start was guided by CD4 thresholds [1, 14, 15]. Subsequent studies predicted factors associated with good HRQoL among PLHIV e.g. being married, absence of co-morbidities, higher education, living in an urban setting, status disclosure, being on ART longer, being employed, fewer pills, and good adherence [16–21]. Conversely, factors found to be associated with lower HRQoL included stigma, same-sex relationships, being symptomatic, illiteracy and not being sexually active [18, 19, 22–24]. HRQoL studies among virally suppressed PLHIV are limited in LMIC [9, 23, 25].

In SSA, HRQoL studies have mostly been conducted among clients who access ART in clinical settings [24, 26–28]. Differentiated service delivery (DSD) is a patient-centered approach which offer virally suppressed PLHIV alternative models of HIV care both within clinic (e.g. multiple month scripting, fast-track refills, adherence/ART clubs etc.) and out-of-clinic (e.g. community ART, community drug distribution points, ART clubs etc.) [29–33]. DSD models benefit both the health system by reducing over-crowding in clinics, improving work efficiency among healthcare workers (HCW), and clients, by fostering self-management, peer support, and reducing time spent seeking care. Out-of-clinic DSD models limit contact with the formal health system and rely upon community health workers (CHW) who are trained volunteers for service delivery. Most evaluations of such models focused on adherence and quality of care yet change in delivery models of care may also affect HRQoL.

With 1.6 million PLHIV and a prevalence of 4.6% among adults in 2018, it was estimated that only 62% of PLHIV on ART in Tanzania are virally suppressed [34]. Though studies show favorable patient-related outcomes with DSD interventions elsewhere, there is a dearth of evidence within the Tanzanian context [35–37]. Additionally, it was not clear how limited contact with the health system, more peer support, less frequent travels impacted the QoL of clients. Our study therefore aimed to assess HRQoL among stable ART clients accessing ART care in a flagship Test and Treat (T&T) project in north-western Tanzania. We compared HRQoL scores and determinants of HRQoL between stable ART clients receiving either standard clinic-based care or ART clubs DSD care.

## Methods

### Study setting and population

The T&T project is hosted by the Catholic Diocese of Shinyanga which covers both Shinyanga and Simiyu regions in north-western Tanzania. Besides Shinyanga urban, Kahama urban, and Bariadi districts, the regions are largely rural. Project sites are four HIV care and treatment centers (CTC) referred to as hubs, two hubs each in the Shinyanga (Ngokolo and Bugisi) and Simiyu (Songambele and Mwamapala) regions. Standard of HIV care in Tanzania is clinic-based and includes one clinic visit every one to three months for consultation, health screening, routine labs and ART refill. DSD in ART clubs was rolled out in the T&T project from July 2018, details of which have been described elsewhere [38]. Briefly, ART clubs are CHW managed groups of 15–30 clients living within the same locality who meet every 3 months for routine health screening and ART distribution. Club members have a clinical consultation every year. Data were collected from May to August 2019. Participants were recruited at the two hubs in the Shinyanga region and their related ART clubs. Eligibility criteria included being adults ≥ 18 years and stable on ART according to the Tanzanian guidelines: on ART 1^st^ line regimen ≥ 6-months, viral load < 50 copies/ml, and no current chronic illness [39]. At the hubs, participants were randomly sampled from a list of all eligible clients who had a clinic appointments within the data collection period. Eligible participants were approached as they attended clinic appointments. All clubs that had a meeting during the data collection period and were at least 6 months or older, were visited. At the clubs, all members were approached as stability was an eligibility criterion for DSD participation. Those clients who gave written consent, completed the interviewer-administered questionnaire.

### Data collection

We used an HIV-specific HRQoL tool that has been validated for the low literacy Swahili population, the Kiswahili translation of the Functional Assessment of HIV Infection (FAHI) [40]. Outcomes of interest were the total and domain-specific FAHI scores. The FAHI is a 47-item tool with five domains namely physical wellbeing (PWB) – 10 items, emotional wellbeing (EWB) – 10 items, functional & global wellbeing (FGWB) – 13 items, social wellbeing (SWB)-8 items, and cognitive functioning (CF) – 3 items [41]. Scores ranged for each item between 0 and 4. We derived (a) domain scores by summing respective item scores – ranges for PWB and EWB were 0 to 40, FGWB 0 to 52, SWB 0 to 32, and CF 0 to12; (b) total FAHI scores by summing all five domain scores – ranging between 44 and 176—note that three items in the PWB domain were not scored as recommended by the FAHI scoring document [42]; (c) FAHI proportional score by calculating each individual score as a proportion of the maximum possible total or domain scores; and (d) a dichotomous (satisfactory/less than satisfactory FAHI HRQoL) variable for total and domains. We considered a score in the highest quintile i.e. ≥ 80% of FAHI total or domain scores as satisfactory to capture all participants who report at least ≥ 4 on the 5-point FAHI tool. This represents all those who report at least above “Somewhat” (i.e. 3 – the midpoint) for all items in all domains of the FAHI instrument.

Secondary outcomes were factors associated with satisfactory HRQoL and domain scores. Three categories of additional data were collected to assess these factors: socio-demographic (location, sex, age, educational level, marital status, employment status, and income level), HIV care (duration on ART, CD4 count at ART start and recency of viral load result) and service access (location, time spent during clinic visit/club meeting[wait time], time spent traveling to clinic/club[travel time] and frequency of service delivery). Data entry, collation, and cleaning were done using EpiData [43].

### Sample size and statistical analysis

Our sample size calculation was based on EQ-index scores and extrapolated to proportional FAHI scores. We assumed a difference in proportional scores of 0.10 (0.80 to 0.90) between the clinic and DSD participants, a standard deviation of 0.40 as determined by Louwagie et al. in South Africa, and a 10% refusal rate requiring thus a minimum of 542 participants overall with 271 participants per service delivery group to have 80% power to reject the null hypothesis of no difference [1].

Categorical variables were presented as percentages and continuous variables as means (± standard deviation) or medians (± interquartile range) as appropriate. Comparisons between clinic and DSD participants were done using Mann Whitney or Kruskal Wallis tests. Association between socio-demographic, HIV care, and service access factors and satisfactory FAHI HRQoL were examined using logistic regression. Sex, age, marital status, and variables showing significant bivariate association at the p-value of < 0.1 were included in the multivariable model. A 3-step hierarchical multiple linear regression was used to quantify the contribution of the three-factor categories to the variance of FAHI scores observed. Socio-demographic variables were entered in the model in the first step, followed by HIV care variables and lastly, service access variables.

We examined variables for multicollinearity using tolerance values and variance inflation factor (VIF) statistics. We generated a Receiver Operative Characteristic (ROC) i.e. area under the curve (AUC) to test the discriminative ability of the model (with all covariates included) to categorize observations as satisfactory/less than satisfactory HRQoL. We assessed the 33 and 28 missing observations dropped from the clinic and DSD in step 3 hierarchical linear models, respectively, to observe any significant differences in mean FAHItotal. All analyses were performed using STATA software version 16.0.

Ethical approval for the study was obtained from the National Institute for Medical Research (NIMR; approval number NIMR/HQ/R.8c/Vol. I/674).

## Results

### Characteristics of the study population

Of 667 PLHIV approached to participate, 641 consented to participate (response rate of 96.1%), and 629 were included in the final analysis (12 excluded due to missing data). While the overall majority of participants were female (63%), there were significantly more men in clinic-based care compared to DSD, and DSD participants were also significantly older (see Table 1). The mean numbers of years-on-ART and mean CD4 count at ART start were significantly longer (4.9 vs 4.1 years, *p* < 0.001) and higher (398.1 vs 341.4 cells/mm^3^, *p* < 0.001) for DSD participants. They also spent shorter time on travel (84.7 vs 34.3 min, *p* < 0.001) and during club meetings (140.2 vs 83.8 min, *p* < 0.001). There were more DSD participants in the urban area (60.6% vs 39.4%). Table 1 provides details on the characteristics of study participants according to the service delivery model.

**Table 1:** Socio-demographic, HIV care, and service access-related characteristics according to service delivery model

	Clinic-based (*n* = 378)	DSD (*n* = 251)	*p*-value*
**Sociodemographic information**			
Location, *n* (%)			
Bugisi (Rural)	324 (65.8)	168 (34.1)	< 0.001
Ngokolo (Urban)	54 (39.4)	83 (60.6)	
Sex (*n*, %)			
Female	224 (59.3)	172 (68.5)	0.018
Male	154 (40,7)	79 (31.5)	
Age in years, median (IQR)	39.3 (33.3–48.1)	44.7 (37.6–54.0)	< 0.001
Age-groups, *n*, (%)			
< 25	25 (6.61)	6 (2.40)	< 0.001
25–34	96 (25.1)	35 (13.9)	
35–44	137 (36.2)	91 (36.3)	
45–54	75 (19.8)	62 (24.7)	
55–64	33 (8.73)	40 (15.9)	
≥ 65	13 (3.4)	17 (6.8)	
Educational level, *n* (%)			
No education	97 (25.7)	60 (23.9)	0.744
Primary	261 (69.1)	180 (71.7)	
≥ Secondary	20 (5.3)	11 (4.4)	
Marital status (*n*, %)			
Single	94 (24.9)	80 (31.9)	0.092
Married	144 (38.1)	78 (31.1)	
Separated/Divorced/Widowed	140 (37.0)	93 (37.1)	
Employment status (*n*, %)			
Unemployed	53 (14.0)	60 (23.9)	0.002
Income level (TSH), median (IQR)	87,000 (50,000- 172,000)	80,000 (50,000- 150,000)	
< 100,000	206 (54.5)	148 (59.0)	0.315
100,000–300,000	116 (30.7)	63 (25.1)	
> 300,000	56 (14.8)	40 (15.9)	
**HIV care information**			
Years on ART, median [IQR]	4.1 [2.1–5.8]	4.9 [2.2–7.3]	0.001
Years on ART group around the mean			
≤ 4.4 years	219 (57.9)	130 (51.8)	0.316
> 4.4 years	150 (39.7)	114 (45.4)	
Missing	9 (2.38)	7 (2.79)	
CD4 at ART start in cells/mm^3^, median [IQR]	341.4 [155–449]	398.1 [184.5–513.5]	0.003
CD4 at ART start groups			
< 200	126 (33.3)	63 (25.1)	0.07
≥ 200	236 (62.4)	173 (68.9)	
Missing	16 (4.23)	15 (5.98)	
Viral load in copies/ml, median [IQR]	10 [10]	10 [10]	0.876
Viral load group			
< 50 copies/ml	375 (99.2)	237 (94.4)	< 0.001
≥ 50	-	9 (3.6)	
Missing	3 (0.8)	5 (2.0)	
Time since last VL record, n (%)			
≤ 6 months	179 (47.4)	113 (45.0)	< 0.001
6 months – 1 year	170 (44.9)	85 (33.9)	
> 1 year	26 (6.9)	49 (19.5)	
Missing	3 (0.8)	4 (1.6)	
**Service access information**			
Time spent in clinic/club in minutes, median [IQR]	140.2 [60–180]	83.8 [30–120]	< 0.001
Length of stay, *n* (%)			
Short (≤ 1 h 30 min)	129 (34.1)	177 (70.5)	< 0.001
Long (> 1 h 30 min)	246 (65.1)	71 (28.3)	
Missing	3 (0.8)	3 (1.2)	
Time spent traveling to clinic/club in minutes, median [IQR]	84.7 [30–120]	34.3 [10–30]	< 0.001
Travel time group, n (%)			
Short (≤ 1 h)	214 (56.6)	232 (92.4)	< 0.001
Long (> 1 h)	161 (42.4)	17 (6.8)	
Missing	3 (0.8)	2 (0.8)	
Frequency of visits/meetings, n (%)			
More (≤ every 2 months)	355 (93.9)	121 (48.2)	< 0.001
Less (> every 2 months)	23 (6.1)	130 (51.8)	

*p-values presented are calculated using Mann Whitney U or Kruskal Wallis tests as appropriate. *n* number, *%* percentage, *SD* standard deviation, *TSH* Tanzanian shilling, *IQR* interquartile range, *ART* antiretroviral treatment, *VL* viral load

### FAHI total and domain scores by service access model

**Fig. 1:**
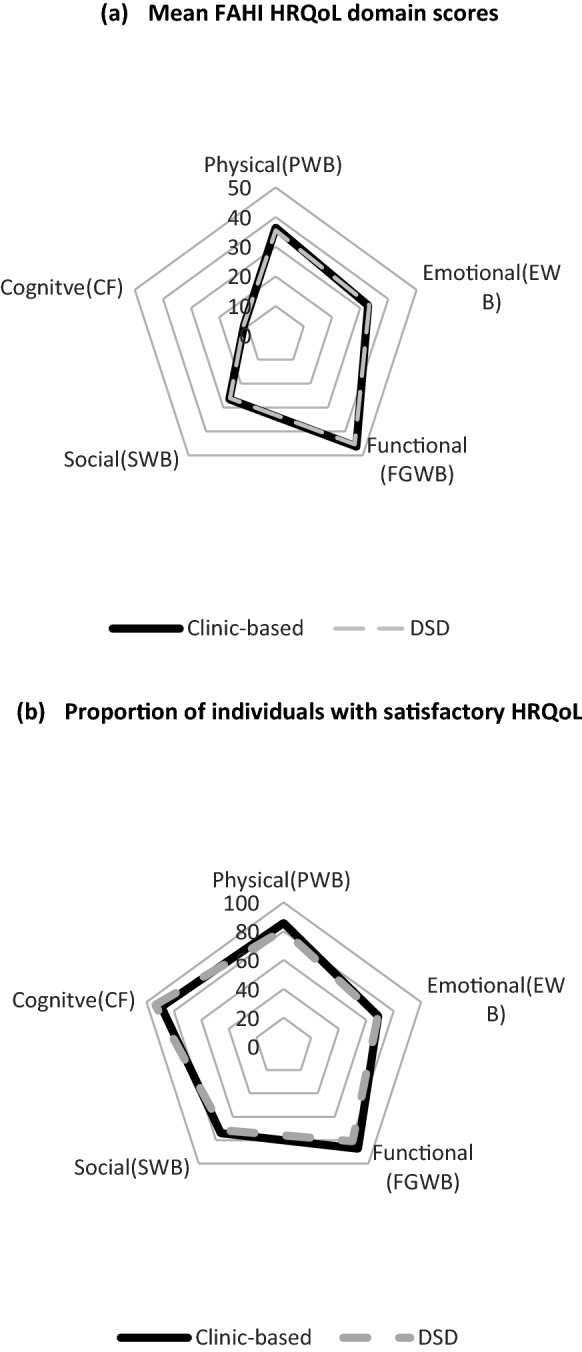
FAHI HRQoL scores by service access model. Mean FAHI HRQoL domain scores (b) Proportion of individuals with satisfactory HRQoL. *PWB* Physical wellbeing, *EWB* Emotional wellbeing, *FGWB* Functional and Global wellbeing, *SWB* Social wellbeing, *CF* Cognitive Functioning

Clinic and DSD participants show comparable mean HRQoL scores across domains with only slight differences in the physical and emotional wellbeing domains (36.4 vs 35.5, max-40 *p* < 0.01) and (32.1 vs 32.8, max-40 *p* < 0.05) (Fig. 1a). No differences were observed in satisfactory HRQoL percentages across domains except for FGWB where more clinic participants revealed satisfactory HRQoL as compared to DSD. Satisfactory HRQoL overall was highest in the CF domain (89.2 vs 93.6) and lowest in the EWB (68.8 vs 68.5) and SWB (74.1 vs71.7) domains (Fig. 1b).

### Associations between sociodemographic, HIV care, and service access factors and satisfactory overall HRQoL

Satisfactory overall HRQoL was associated with being male ((odds ratio 2.59, 95% confidence interval 1.36–4.92) among clinic participants and with living in an urban setting (4.72, 1.70–13.1) in DSD care (see—Table 2). Less than satisfactory HRQoL was observed with increasing age among clinic participants, and with increasing income, and increased meeting duration among DSD participants. HIV care factors were generally not associated with satisfactory overall HRQoL.

**Table 2:** Logistic regression with robust variance: Multivariable association between sociodemographic, HIV care, and service access variables and satisfactory FAHI QoL scores#

	FAHItotal		PWB		EWB		SWB	
	Clinic	DSD	Clinic	DSD	Clinic	DSD	Clinic	DSD
Sociodemographic	Odds ratios and confidence intervals							
Sex	Ref.	Ref.	Ref.	Ref.	Ref.	Ref.	Ref.	Ref.
Female	2.59**	1.11	1.02	0.71	1.39	0.87	1.98*	1.95
Male	1.36–4.92	0.49–2.51	0.51–2.04	0.31–1.62	0.83–2.33	0.36–2.12	(1.14–3.43)	(0.89–4.27)
Age								
18–25	Ref.	Ref.	Ref.	Ref.	Ref.	Ref.	Ref.	Ref.
25–35	0.16	1.15	0.17	2.12	0.61	0.18	0.33	1.32
	0.02–1.43	0.01–22.62	0.02–1.55	0.11–39.4	0.20–1.83	0.00–1.45	(0.08–1.34)	(0.11–15.78)
35–45	0.0.09*	1.33	0.17	2.86	0.62	0.43	0.21*	1.19
	0.01–0.78	0.07–23.91	0.02–1.52	0.18–46.3	0.21–1.84	0.00–3.28	(0.05–0.84)	(0.11–12.91)
45–55	0.06*	0.62	0.16	1.29	0.49	0.16	0.26	0.67
	0.01–0.59	0.03–11.3	0.02–1.56	0.08–20.7	0.15–1.58	0.00–1.27	(0.06–1.09)	(0.06–7.40)
55–65	0.08*	0.55	0.25	1.78	0.81	0.14	0.33	1.08
	0.01–0.85	0.03–10.5	0.02–2.82	0.10–31.3	0.21–3.05	0.00–1.11	(0.07–1.63)	(0.09–12.82)
> 65	0.04*	0.36	0.05*	0.58	0.58	1.19	0.35	0.76
	0.00–0.52	0.02–7.96	0.00–0.59	0.03–11.2	0.11–2.98	0.01–1.17	(0.06–2.12)	(0.05–10.43)
Education								
None	Ref.	Ref.	Ref.	Ref.	Ref.	Ref.	Ref.	Ref.
Primary	1.18	0.8	0.74	0.65	0.98	0.87	1.33	0.23**
	0.62–2.20	0.33–1.93	0.35–1.52	0.26–1.61	0.57–1.69	0.32–2.36	(0.76–2.31)	(0.09–0.58)
≥ Secondary	0.43	1	0.31	1	0.77	1	0.96	0.59
	0.12–1.60		0.07–1.32		0.24–2.52		(0.28–3.30)	(0.05–6.44)
Marital status								
Single	Ref.	Ref.	Ref.	Ref.	Ref.	Ref.	Ref.	Ref.
Married	1.98	2.22*	2.22*	3.92*	1.77	1.49	4.08**	4.93**
	0.96–4.07	0.89–5.50	1.01–4.89	1.37–11.2	0.95–3.30	0.53–4.12	(2.11–7.87)	(2.06–11.75)
Separ/Divorc/Wid	1.72	1.53	1.54	1.53	1.26	0.73	1.91*	6.35**
	0.85–3.49	0.62–3.79	0.71–3.30	0.63–3.76	0.68–2.33	0.26–2.00	(1.04–3.52)	(2.60–15.53)
Employment								
Unemployed vs Employ			Ref.	Ref.				
			1.44	0.39				
			0.58–3.56	0.14–1.14				
Income level								
< 100,000	Ref.	Ref.			Ref.	Ref.		
100,000–300,000	0.45*	0.43			0.48**	0.6		
	0.24–0.84	0.18–1.02			0.28–0.82	0.22–1.62		
> 300,000	1.09	0.28*			0.46*	0.23*		
	0.43–2.79	0.11–0.78			0.23–0.91	0.07–0.77		
Location								
Bugisi	Ref.	Ref.	Ref.	Ref.	Ref.	Ref.	Ref.	Ref.
Ngokolo	1.88	4.72**	3.05	3.94*	4.81**	17.1**	0.94	3.79**
	0.74–4.81	1.70–13.13	0.94–9.89	1.34–11.5	1.86–12.42	4.63–62.8	(0.45–1.98)	(1.62–8.86)
Wait time mins	Ref.	Ref.	Ref.	Ref.	Ref.	Ref.	Ref.	Ref.
< 90 min	0.9	0.23**	0.89	0.43*	0.64	0.05**	0.75	0.24**
> 90 min)	0.47–1.73	0.11–0.47	0.44–1.79	0.21–0.92	0.37–1.10	0.02–0.12	(0.42–1.32)	(0.12–0.48)

*p < 0.01; **p < 0.001. # See Additional file 1 for table with FGWB and CF domain results.

### Associations between sociodemographic, HIV care and service access factors and satisfactory domain HRQoL

Compared to being single, satisfactory HRQoL was associated with being married in the PWB domain for both clinic and DSD. Being married or separated, divorced, or widowed was positively associated with satisfactory HRQoL for both clinic and DSD participants in the SWB domain and only among DSD participant in the FGWB domain. Living in an urban area was significantly associated with satisfactory HRQoL for both clinic and DSD participants in the EWB domains and only among DSD participants in the SWB and PWB domains. Across domains, declining age was generally not associated with satisfactory HRQoL. Significantly less satisfactory HRQoL was only seen among clinic participants aged over 65 years in the PWB and FGWB domains. Surprisingly, less satisfactory HRQoL was linked with increased income levels in the EWB domain among clinic and DSD participants. Generally, HIV care factors were not associated with satisfactory HRQoL. Among service access factors, DSD participants alone reported less than satisfactory HRQoL for spending longer time during service access in the PWB, EWB, and SWB domains (Table 2 and Additional file 1 [for additional results of FGWB & CF domains]).

### Contribution of sociodemographic, HIV care, and service access factors to variance observed in HRQoL.

Table 3 shows the contribution of sociodemographic, HIV care, and service access factors to the variance observed in HRQoL scores. The analyses revealed that among clinic participants, the variance in overall HRQoL score FAHI total explainable by sociodemographic variables in the first step was 10.2%. The addition of HIV care variables in the second step increased the variance explained to 14.5%. Finally, service access variables in the third step brought the total to 14.9%. For DSD participants, the variance explained was 22.9%, 28.9%, and 43.5% in the first, second, and third steps, respectively. Across all domains, the variance in HRQoL explainable by the 3-step hierarchical model for clinic participants was modest (see Table 3). The highest was reported in the EWB domain i.e. 8.5%, 11.4%, and 18.5%, and lowest in the CF domain i.e. 5.9%, 9.1%, and 9.8%, respectively. A much higher proportion of variance was explained in overall FAHI 43.5%, PWB 30.2%, EWB 53.4%, and SWB 35.1% among DSD participants. Additional file 2 shows the details of the hierarchical linear regression with coefficients of all covariate in each step.

**Table 3:** Contribution of sociodemographic, HIV care, and service access factors to variance observed in HRQoL scores

Variance explained by three-factor categories (N = clinic vs DSD)												
	FAHItotal		PWB		EWB		FGWB		SWB		CF	
	Clinic	DSD	Clinic	DSD	Clinic	DSD	Clinic	DSD	Clinic	DSD	Clinic	DSD
*Step 1 R^2^ (*n* = 378 vs 251)	0.102	0.229	0.103	0.205	0.085	0.184	0.119	0.222	0.118	0.217	0.059	0.058
^Step 2 R^2^ (*n* = 351 vs 226)	0.145	0.289	0.146	0.243	0.114	0.268	0.169	0.253	0.139	0.258	0.091	0.086
^#^Step 3 R^2^ *n* = 345 vs 223	0.149	0.435	0.149	0.302	0.185	0.534	0.167	0.297	0.148	0.351	0.098	0.099
AIC Step 3	8.803	8.692	5.969	6.077	6.603	6.415	6.258	6.63	6.723	6.523	4.034	4.081

*Step 1—Contribution of sociodemographic factors to variance observed; ^Step 2—Contribution of HIV care factors to variance observed and ^#^Step 3 – Contribution of service access variables to variance observed from Hierarchical Multiple Linear Regression. Additional file 2 shows the regression coefficients for variables included in the models in steps 1–3.

## Internal consistency and Goodness of fit statistics

In the present study, Cronbach alpha was 0.68, 0.73, 0.67, 0.71, and 0.81 for the PWB, EWB, FGWB, SWB, and CF domains, respectively, indicating acceptable internal consistency. Tolerance values ranged from 0.16 to 0.84 while the VIF values were from 1.19 to 6.3 suggesting that multicollinearity had no impact on the variables included. The AUC for our logistic regression model was 0.81 showing the acceptable ability of our model to discriminate – the effective range is usually from 0.5 to 1. There was no significant difference in mean FAHI total scores when the step 3 models in the hierarchical regression were compared with step 1 models.

## Discussion

Our study compared factors influencing HRQoL among stable ART clients accessing care at either HIV clinics or DSD clubs in the Shinyanga region of Tanzania. Most participants in our study rate their HRQoL as satisfactory. Our results revealed that service access factors contributed considerably to HRQoL among DSD participants. We found that time spent during clinic/club and the settings of service delivery were factors significantly associated with perceived HRQoL.

Understanding HRQoL in African studies is relevant in the current era of expanded treatment” and DSD. Previous HRQoL studies compared HIV positive and negative people and/or PLHIV not on and on ART [2, 2]. Similar HRQoL among stable clients seen in our study strengthens the case for DSD which may likely impact positively on care delivery to unstable clients concurrently who are more likely to have special needs [44, 45]. The complex effect of service access factors on overall HRQoL suggests that other non-measured factors are likely also to influence HRQoL.

Service access factors are more commonly studied about patient satisfaction and retention in care than in HRQoL but both are likely to be related. The shorter time spent accessing service observed as positively associated with HRQoL in our study may reflect the value placed on other meaningful engagements made possible by the time saved from care seeking in this setting. Being predominantly farmers, reduction of productivity loss due to care seeking likely impacts HRQoL. In Malawi and Uganda, reduced time spent in DSD models was reported as a favorable outcome predicting retention and satisfaction [46, 47]. Reduced travel time has also been identified as beneficial for DSD participants and enabling its success, although it was not independently associated with HRQoL in our study [48, 49].

As per HRQoL domains, the literature reveals that social and psychological/emotional domains score the lowest in most HRQoL studies among PLHIV [16, 21, 25, 50–54] which is in line with our findings. Reasons adduced for this include stigma and discrimination due to fear and lack of awareness as HIV continues to isolate those infected from meaningful relationships. The slight difference in the PWB domain scores is likely not clinically significant as HRQoL was generally not associated with most covariates except for those age > 65 years or married in the clinic. The variance explainable due to service access factors was notably largest i.e. 53.4% in the EWB domain suggesting some significance of the contribution of DSD in supporting participants who likely face different psychological, emotional, and social dilemmas. [16, 17, 51].

While our finding that being male was associated with a more satisfactory HRQoL aligns with evidence from Tanzania, Burkina Faso, Ghana, and Ethiopia [18, 55–57], other studies reveal either no association [23, 25, 58] or favor higher HRQoL among women [22, 50, 59]. Although these studies did not target stable clients, they illustrate the complexity of associations between gender and HRQoL. We note across studies that women living in male dominated settings (as is the case in our study) tend to report lower HRQoL when compared with settings where women have social support.

Similar to findings with gender, age reveals intricacies of associations in literature, showing evidence of declining HRQoL with age [54, 55, 57, 59] among PLHIV, as well as improvement or no association [18, 23, 60]. Given that DSD participants in our study were significantly older, our finding a trend of declining HRQoL with age mainly among clinic participants suggests a protective effect of DSD on HRQoL with increasing age. Older adults may enjoy fewer social ties than younger adults and thus reap a larger emotional benefit from DSD. As the PLHIV population on ART ages and comorbidities increase, emotional support will become increasingly important and DSD could serve as a springboard for additional interventions.

Context such as place of residence has been associated with HRQoL in LMIC [20, 21]. Our study showed that urban participants had higher HRQoL scores across most domains than did their rural counterparts. Better living conditions, greater awareness about HIV, and the anonymity people generally enjoy living in an urban setting likely creates a less-stigmatizing space for PLHIV. Our findings that educational level, employment, and income level was not associated with HRQoL however differs from reports in the literature which associates a better HRQoL among PLHIV with a higher level of education [18, 20, 53–55]; with employment [19, 59, 61] and relatedly to higher income levels [19, 62]. The prevailing socio-economic circumstances which are similar among participants irrespective of setting could provide an explanation.

Despite viral suppression, HIV infection predicts sup-optimal HRQoL [9, 25]. The assumption of ‘normalcy’ in all areas as PLHIV attain viral suppression may be ambitious especially in the context of stigma, living in socio-economically difficult circumstances, or with other chronic illnesses. The need to do more in these areas has been advocated especially for PLHIV in the rural areas, for women, and adolescents, and young people living with HIV [18, 24, 25, 61, 63].

### Strengths and limitations

Our study is among few HRQoL studies conducted recently in SSA in the era of DSD. It provides useful insights into factors influencing HRQoL in an African population. Our participants were drawn from different geographical settings that mimic the reality of our population and generated valuable information about the impact of DSD in these settings. Though observational with known biases, the analytical design of our study allowed for comparisons that produced a rich resource useful for informing implementation and policy.

Clinic participants were selected for stability as defined by the Tanzanian guideline at the time of data collection while DSD participants were assumed to be stable. This might have biased our results in favor of clinic participants, however, viral load-related variables were similar in both groups and not independently associated with HRQoL in our study.

The project sites were mission clinics which may limit the generalizability of our findings. However, we might expect that larger differences in HRQoL scores would be found when comparing DSD and clinics outside the mission hospital setting, as better funding and service which characterize our setting likely obscured the effect of DSD.

## Conclusion

Our results reveal comparable HRQoL between clinic and DSD participants. The similarity was also observed across HRQoL domains only differing in the PWB and EWB domains where clinic participants score higher. Better HRQoL was associated with being male among clinic participants and with being married, urban residence and shorter duration of wait during service access among DSD participants. While DSD shows promise in improving acceptability among clients and, therefore, the sustainability of such services, our research highlights future areas to explore to further improve HRQoL among PLHIV. Service providers will need to engage PLHIV and the community at large to identify supporting interventions relevant for adapting acceptable DSD interventions to maximize their benefit.

## Supplementary Information

Below is the link to the electronic supplementary material.

Supplementary file1 (XLSX 16 KB)

Supplementary file2 (XLSX 24 KB)

## Data Availability

The dataset used and analyzed during the current study are available from the corresponding author on reasonable request.

## Abbreviation

ART: Anti-Retroviral Therapy
ARV: Antiretroviral
AUC: Area Under Curve
AYPLHIV: Adolescent and Young People Living with HIV
CF: Cognitive Functioning
CI: Confidence Interval
CHW: Community Health Worker
CTC: Care and Treatment Centre
DSD: Differentiated Service Delivery
EWB: Emotional Wellbeing
FAHI: Functional Assessment of HIV Infection
FGWB: Functional and Global Wellbeing
HIC: High Income Country
HIV: Human Immunodeficiency Syndrome
HCW: Health Care Worker
HRQoL: Health-Related Quality of Life
LMIC: Low- and Middle-Income Country
NACP: National AIDS Control Program
NIMR: National Institute for Medical Research
MOHCDGEC: Ministry of Health, Community Development, Gender, Elderly, and Children Tanzania
PLHIV: People Living with HIV
PWB: Physical Wellbeing
QoL: Quality of Life
ROC: Receiver Operative Characteristics
SD: Standard Deviation
SSA: Sub-Saharan Africa
SWB: Social Wellbeing
T & T: Universal Test & Treat
VL: Viral Load
VLS: Viral Load Suppression
WHO: World Health Organization
